# Controlling chloride ions diffusion in concrete

**DOI:** 10.1038/srep03359

**Published:** 2013-11-28

**Authors:** Lunwu Zeng, Runxia Song

**Affiliations:** 1 Jiangsu Key Laboratory for Intelligent Agricultural Equipment, College of Engineering, Nanjing Agricultural University, Nanjing 210031, China

## Abstract

The corrosion of steel in concrete is mainly due to the chemical reaction between the chloride ions and iron ions. Indeed, this is a serious threaten for reinforced concrete structure, especially for the reinforced concrete structure in the sea. So it is urgent and important to protect concrete against chloride ions corrosion. In this work, we report multilayer concrete can cloak chloride ions. We formulated five kinds of concrete A, B, C, D and E, which are made of different proportion of cement, sand and glue, and fabricated six-layer (ABACAD) cylinder diffusion cloak and background media E. The simulation results show that the six-layer mass diffusion cloak can protect concrete against chloride ions penetration, while the experiment results show that the concentration gradients are parallel and equal outside the outer circle in the diffusion flux lines, the iso-concentration lines are parallel outside the outer circle, and the concentration gradients in the inner circle are smaller than those outside the outer circle.

In 2006, Pendry designed perfect invisibility cloak^1^. Following this method, varieties of electromagnetic cloak^2^,^3^,^4^,^5^,^6^,^7^,^8^,^9^,^10^,^11^,^12^,^13^,^14^,^15^,^16^,^17^, acoustic cloak^18^,^19^,^20^,^21^,^22^,^23^, mater wave cloak^24^, magnetic cloak^25^,^26^,^27^, dc electric cloak^28^,^29^, heat cloak^30^,^31^,^32^,^33^,^34^,^35^,^36^,^37^,^38^ and mass diffusion cloak^39^ have been designed. The first experiment^4^ demonstrated invisible cloak having adopted metamaterial with simplified material parameters, the simplified cloak inherits some properties of the ideal cloak, but finite scatterings exist. Perfect invisible cloak requires inhomogeneous and anisotropic media, however, it is difficult to construct by using natural materials. Recently, a cylinder heat cloak structure that does not require metamaterials to realize the anisotropy and inhomogeneity of the material parameters was proposed^32^; it was realized by a concentric layered structure consisting of alternating homogeneous isotropic materials, which can be treated as an effective medium with the radius dependent anisotropy. The time-dependent thermodynamic problem has also been treated theoretically^33^ and experimentally^37^ by Guenneau and co-author. Subsequent Guenneau^39^ adapted the concept of transformational thermodynamics, the n-dimensional, time-dependent, anisotropic heterogeneous Fick's equation is derived. Based on the above research, we fabricated six-layer cylinder mass diffusion cloak according to the diffusion theory. The experiment demonstrated that the six-layer structures can also cloak diffusion flux. Our work proves the feasibility to extend the electromagnetic transformation optic devices into the mass diffusion area, which may pave a way to control the diffusion flux for novel device applications.

## Results

### Mass diffusion principle

The mass diffusion equation can be written as: 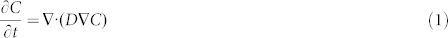
where *t* is time, *C* is mass diffusion concentration (*mol* · *m*^−3^ or *kg* · *m*^−3^, et. al), *D* is diffusion coefficient (*m*^2^ · *s*^−1^), 
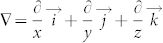
 is the operator in the Cartesian coordinate system. Generally speaking, the mass diffusion concentration is the function of space and time coordinate (*x*, *y*, *z*, *t*), i.e., *C* = *C* (*x*, *y*, *z*, *t*). According to the mass diffusion theory, we can obtain the following simplified radial and tangential mass diffusion coefficient similar thermal diffusion coefficient^33^,^37^

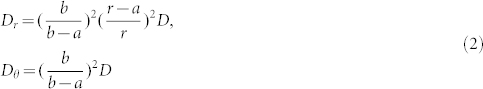
The cylindrical anisotropy can be obtained by layered structure of homogeneous isotropic materials^33^. Assumed that two kinds of materials have a homogeneous and an isotropic diffusion coefficients *D_A_* and *D_B_*, respectively, the thicknesses are *d_A_* and *d_B_*, respectively, we obtain the following effective parameters *D_r_* and *D_θ_*

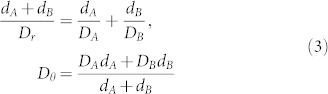
where *D_r_* is the radial diffusion coefficient, and *D_θ_* is the tangential diffusion coefficient.

**Figure 1:**
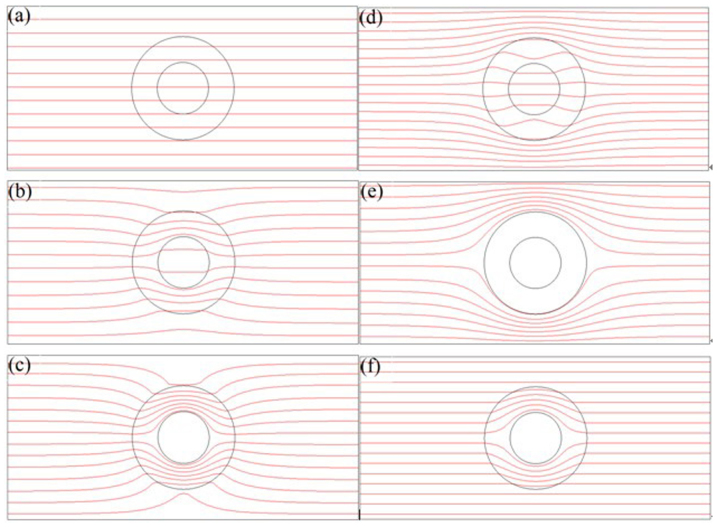
Principle of mass diffusion. (a) The relative diffusion coefficient of the hollow cylinder (D_c_ = 1.0) is equal to the relative diffusion coefficient of the background media (D_b_ = 1.0). (b) The hollow cylinder with large relative diffusion coefficient (D_c_ = 4.0) attracts diffusion flux lines. (c) The hollow cylinder with giant relative diffusion coefficient (D_c_ = 10000) completely attracts diffusion flux lines. (d) The hollow cylinder with small relative diffusion coefficient (D_c_ = 0.25) repels diffusion flux lines. (e) The hollow cylinder with very small relative diffusion coefficient (D_c_ = 0.0001) completely repels diffusion flux lines. (f) Mass diffusion cloak, the diffusion coefficient of the hollow cylinder fulfills Eq. (2).

According to the diffusion theory, we simulate mass diffusion. Fig. 1a shows that the hollow cylinder and the background medium have identical relative diffusion coefficient (D_c_ = D_b_ = 1.0), the diffusion coefficient of the hollow cylinder may be an effective diffusion coefficient of isotropy and homogeneous composite material, the diffusion flux lines are parallel both in the background media and hollow cylinder, this phenomenon is called “mass diffusion transparency”. Fig. 1b shows that the relative diffusion coefficient of the hollow cylinder (D_c_ = 4.0) is larger than that of the background media (D_b_ = 1.0), the diffusion flux lines are attracted by the hollow cylinder, and the diffusion flux lines are distorted both inside and outside the hollow cylinder. Fig. 1c shows that the relative diffusion coefficient of the hollow cylinder (D_c_ = 10000) is much larger than that of the background media (D_b_ = 1.0), the diffusion flux lines are completely attracted by the hollow cylinder, no diffusion flux lines penetrate into the inner circle, this phenomenon is called “mass diffusion shielding”, just like magnetostatic (electrostatic) shielding is generated by large permeability (permittivity) material. Fig. 1d shows the relative diffusion coefficient of the hollow cylinder (D_c_ = 0.25) is smaller than that of the background media (D_b_ = 1.0), the diffusion flux lines are repelled by the hollow cylinder, and the diffusion flux lines are also distorted both inside and outside the hollow cylinder. Fig. 1e shows the relative diffusion coefficient of the hollow cylinder (D_c_ = 0.0001) is much smaller than that of the background media (D_b_ = 1.0), the diffusion flux lines are completely repelled by the hollow cylinder, no diffusion flux lines penetrate into the inner circle, just like magnetic field lines are completely repelled by an “superconductor”. Fig. 1f shows the mass diffusion cloak, the diffusion coefficient of the hollow cylinder fulfills Eq. (2), the diffusion flux lines are parallel outside the outer cylinder, no diffusion flux lines penetrate into the inner circle. Theoretically and experimentally, the conditions of the mass diffusion cloak are that the concentration gradients are parallel and equal outside the outer circle, the iso-concentration lines are parallel outside the outer cylinder, the concentrations and concentration gradients in the inner circle are smaller than those outside the outer circle.

### Simulate steady mass diffusion cloak

**Figure 2:**
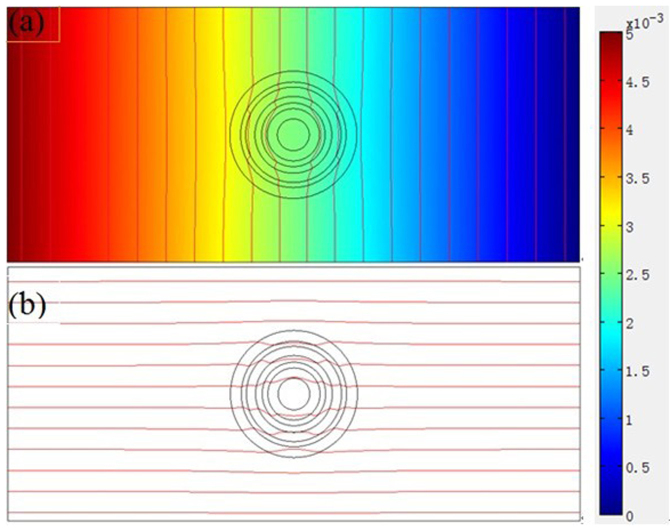
Simulate steady state six-layer diffusion cloak. (a) The surface and the iso-concentration lines. The left is large concentration, the right is small concentration. The vertical lines are the iso-concentration lines. (b) The horizontal lines are diffusion flux lines, and the diffusion flux is opposite to the direction of the concentration gradient.

According to Eq. (2) and Eq. (3), we design six-layer (ABACAD) diffusion cloak and simulate concentration distributions. The inner and outer radii of the six-layer (ABACAD) are (1, 1.403), (1.403, 2), (2, 2.356) (2.356, 3), (3, 3.318), (3.318, 4), respectively, and the diffusion coefficients of the four kinds of materials are D_A_ = 4.000 × 10^−8^ cm^2^/s, D_B_ = 0.278 × 10^−8^ cm^2^/s, D_C_ = 0.547 × 10^−8^ cm^2^/s, D_D_ = 0.741 × 10^−8^ cm^2^/s, respectively, the diffusion coefficient of the background media are D_E_ = 1.000 × 10^−8^ cm^2^/s. Fig. 2 shows the surface, iso-concentration lines and diffusion flux lines of the six-layer diffusion cloak; the vertical lines are iso-concentration lines (Fig. 2a), the red parts represent large concentration region, the concentration is set to be 0.005 mol/m^3^ on the left plane, and the blue parts represent small concentration region, the concentration is set to be 0 on the right plane, the horizontal lines are diffusion flux lines (Fig. 2b). The incident diffusion flux lines are guided around the inner circle, outside the cloaked region, the incident diffusion flux lines return to their original direction as if nothing has happened. No diffusion flux lines penetrate into the inner circle. The diffusion flux is opposite to the direction of the concentration gradient.

**Figure 3:**
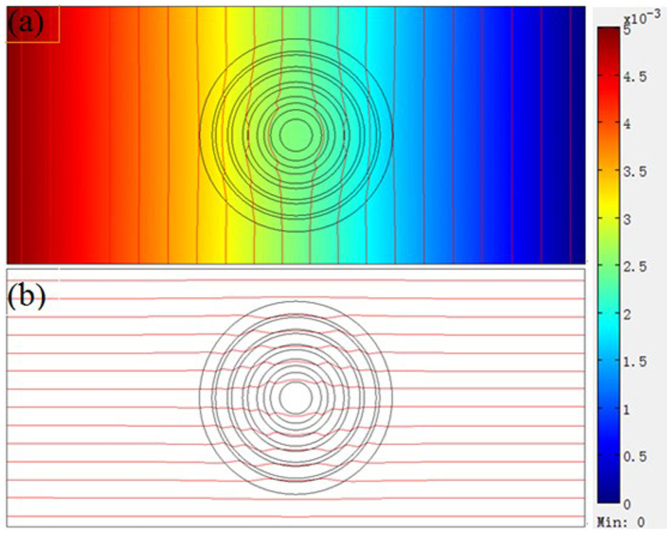
Simulate steady state ten-layer diffusion cloak. (a) The surface and the iso-concentration lines. The left is large concentration, the right is small concentration. The vertical lines are the iso-concentration lines. (b) The horizontal lines are diffusion flux lines; the performance of ten-layer diffusion cloak is better than that of six-layer diffusion cloak.

Fig. 3 shows the ten-layer (ABACADAEAF) diffusion cloak, Fig. 3a shows the surface and iso-concentration lines, Fig. 3b shows diffusion flux lines, the inner and outer radii of the ten-layer (ABACADAEAF) are (1, 1.317), (1.317, 2), (2, 2.271), (2.271, 3), (3, 3.236), (3.236, 4), (4, 4.208), (4.208, 5), (5, 5.186), (5.186, 6), respectively, and the diffusion coefficients of the six kinds of materials are D_A_ = 4.000 × 10^−8^ cm^2^/s, D_B_ = 0.253 × 10^−8^ cm^2^/s, D_C_ = 0.478 × 10^−8^ cm^2^/s, D_D_ = 0.650 × 10^−8^ cm^2^/s, D_E_ = 0.766 × 10^−8^ cm^2^/s, D_F_ = 0.855 × 10^−8^ cm^2^/s, respectively, the diffusion coefficients of the background media are D_G_ = 1.000 × 10^−8^ cm^2^/s. The diffusion coefficient of the background media are D_E_ = 1.000 × 10^−8^ cm^2^/s. Simulation results show that the more the layers, the better performance the mass diffusion cloak, and the small diffusion coefficient materials exclude diffusion flux lines and the large diffusion coefficient materials attract diffusion flux lines, just like small permittivity (permeability) material excludes electric (magnetic) field line and large permittivity (permeability) material attracts electric (magnetic) field line. It should be pointed out that the mass diffusion cloak mainly acts as a flux-guiding shell to suppress the influence of diffusion reflection.

### Simulate time-dependent multilayer diffusion cloak

**Figure 4:**
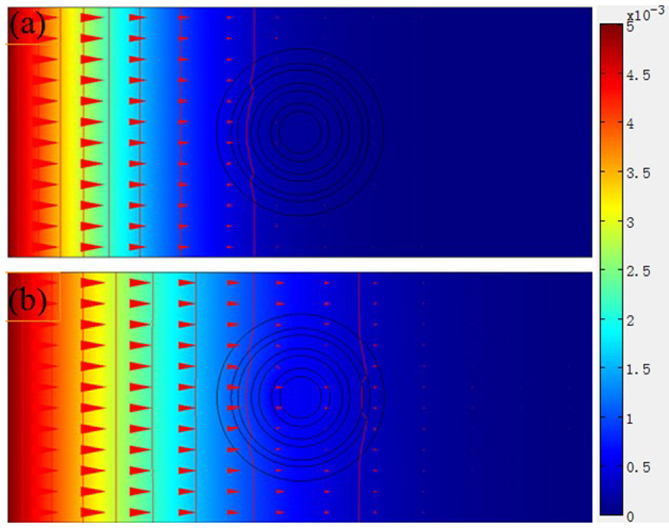
Simulate time-dependent six-layer diffusion cloak. (a) The surface, iso-concentration lines and diffusion flux arrows. The vertical lines are the iso-concentration lines which are parallel outside the outer circle. The horizontal arrows represent diffusion fluxes which are parallel outside the outer circle. The left is large concentration and the right is small concentration. The diffusion time is 100 minutes. (b) The diffusion time is 200 minutes.

**Figure 5:**
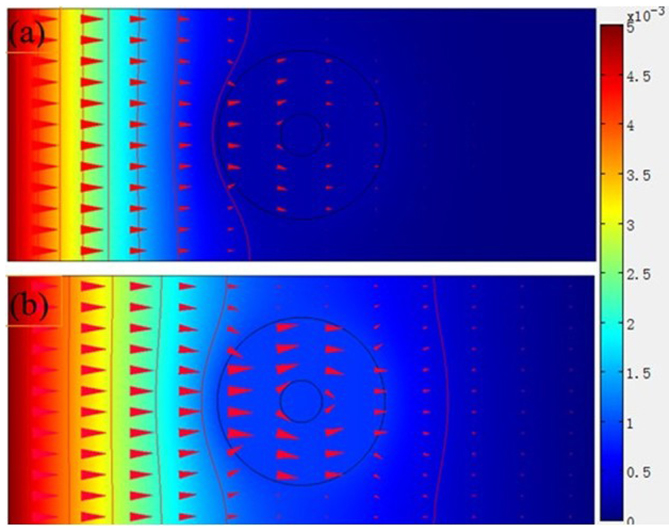
Simulate time-dependent non-cloak. (a) The surface, iso-concentration lines and diffusion flux arrows. The vertical lines are the iso-concentration lines which are not parallel outside the outer circle. The horizontal arrows are diffusion fluxes which are not parallel outside the outer circle. The diffusion time is 100 minutes. (b) The diffusion time is 200 minutes.

Utilizing the transient diffusion equation of COMSOL Multiphysic software, we simulate the diffusion cloak in different time. The parameters are the same as that in Fig. 2. The diffusion time is 100 minutes (Fig. 4a) and 200 minutes (Fig. 4b). Outside the outer circle, the vertical iso-concentration lines are parallel and the horizontal diffusion flux arrows are also parallel. For comparison, we also simulate the non-cloak in different diffusion time. The diffusion time is also 100 minutes (Fig. 5a) and 200 minutes (Fig. 5b). The inner and outer radii of the hollow cylinder are a = 1, and b = 4, respectively. The diffusion coefficients of the background media and hollow cylinder are D_b_ = 1.000 × 10^−8^ cm^2^/s and Dc = 2.500 × 10^−8^ cm^2^/s, respectively. Outside the outer circle, the iso-concentration lines are not parallel and the horizontal diffusion flux arrows are also not parallel. So this hollow cylinder is a non-cloak.

### Experiment realization

**Figure 6:**
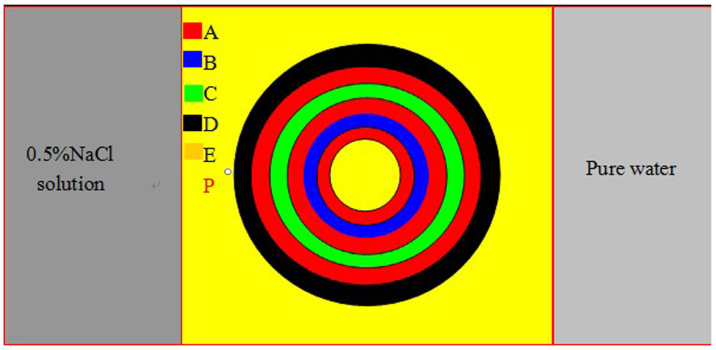
Geometry of the six-layer diffusion cloak. The diffusion cloak is in the center of the sink, the left is 0.5% NaCl solution, the right is pure water. The red, blue, green, black and yellow regions represent concrete A, B, C, D and E, respectively.

**Figure 7:**
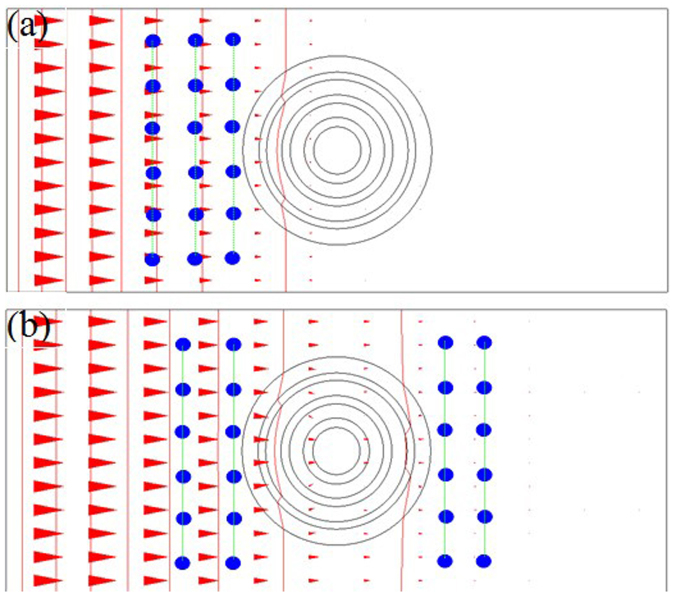
Test points of the time-dependent concentrations in the iso-concentration lines of the six-layer cloak. (a) The parameters and the diffusion time are the same as that in Fig. 4a, the small blue cylinders are located at the iso-concentration lines, and the measurement results of the concentrations are shown in Fig. 8a. (b) The parameters and the diffusion time are the same as that in Fig. 4b, the measurement results of the concentrations are shown in Fig. 8b.

**Figure 8:**
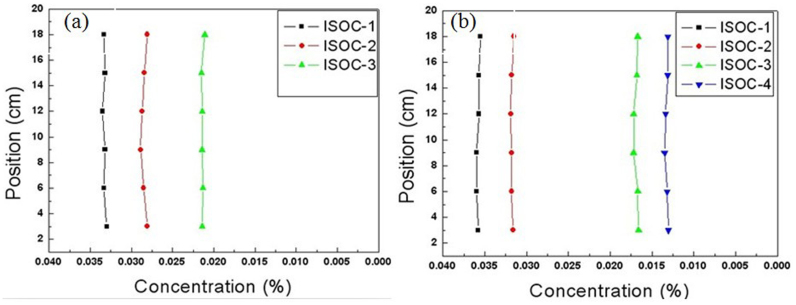
Measurement results of the time-dependent concentrations in the iso-concentration lines of six-layer cloak. (a) The concentrations in three parallel lines. The parameters and the diffusion time are the same as that in Fig. 7a. (b) The concentrations in four parallel lines. The parameters and the diffusion time are the same as that in Fig. 7b.

The geometry of the six-layer diffusion cloak is shown in Fig. 6, the geometric parameters and material parameters in the experiment completely accord with those in simulation. The red, blue, green, black and yellow regions represent concrete A, B, C, D and E, respectively. The diffusion cloak is in the center of the sink, the left is 0.5% NaCl solution, and the right is pure water. The concretes are made of cement, sand and glue. After many times try, we obtain the concretes with various diffusion coefficients we expect. The proportion of cement, sand and glue are about A (1:3:0.02), B (1:3:0.30), C (1:3:0.20), D (1:3:0.10), E (1:3:0.08), respectively. The diffusion coefficients are D_A_ = 4.00 × 10^−8^ cm^2^/s, D_B_ = 0.28 × 10^−8^ cm^2^/s, D_C_ = 0.55 × 10^−8^ cm^2^/s, D_D_ = 0.74 × 10^−8^ cm^2^/s, D_E_ = 1.00 × 10^−8^ cm^2^/s, respectively. According to the measurement results, we find that the more the glue, the smaller the diffusion coefficient, i.e., the glue can decrease diffusion coefficient. It is also proved that other material, such as fly ash, can also decrease diffusion coefficient. According to the simplified parameter Eq. (2) and effective parameter Eq. (3), we fabricate the six-layer (ABACAD) cylinder diffusion cloak. The inner and outer radii of the six-layer cylinder cloak are (1 cm, 1.4 cm), (1.4 cm, 2.0 cm), (2,0 cm, 2.4 cm), (2.4 cm, 3.0 cm), (3.0 cm, 3.3 cm), (3.3 cm, 4.0 cm), respectively, the height of the cylinder cloak is h = 6 cm (in order to measure the mass diffusion in different time, we fabricated many identical diffusion cloaks which are made of the same materials). After 100 minutes diffusion, pasting simulation results (Fig. 4a and Fig. 4b) on the surface of samples, we dug up a lot of cylinders (the diameter is 4 mm) in the vertical straight lines (Fig. 7a), and measured the chloride ions concentration by **NJCL** **Chloride Ions Concentration Fast Measurement Equipment**. The concentrations of 18 test points are shown in Fig. 8a, the concentrations in a straight line are nearly equal and the concentrations in three vertical straight lines (Fig. 8a) are about 0.0330%, 0.0280% and 0.0220%, respectively. So the three vertical straight lines are the iso-concentration lines. After 200 minutes diffusion, we measured the concentrations of 24 test points (Fig. 7b), the concentrations in a straight line are nearly equal and the concentrations in four vertical straight lines (Fig. 8b) are about 0.0360%, 0.0330%, 0.0180% and 0.0130%, respectively. So the four vertical straight lines are the iso-concentration lines.

**Figure 9:**
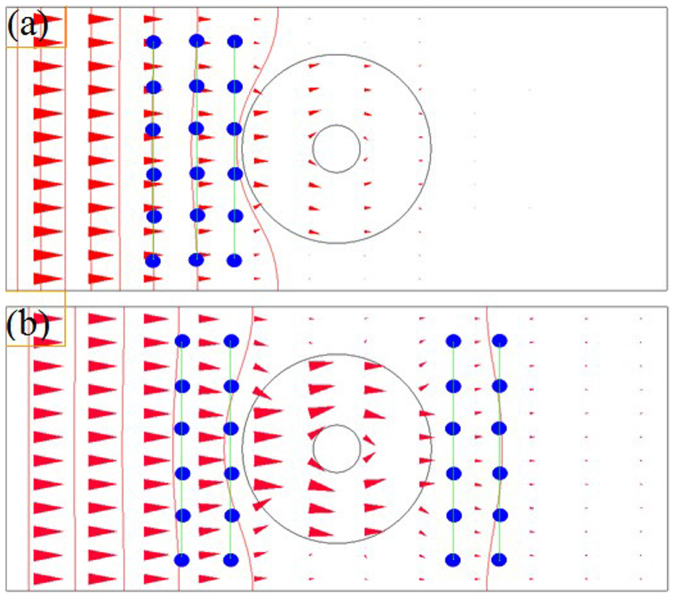
Test points of time-dependent concentrations in the vertical straight lines of non-cloak. (a) The parameters and the diffusion time are the same as that in Fig. 5a, the small blue cylinders are located at the vertical straight lines, and the measurement results of the concentrations are shown in Fig. 10a. (b) The parameters and the diffusion time are the same as that in Fig. 5b, the measurement results of the concentrations are shown in Fig. 10b.

**Figure 10:**
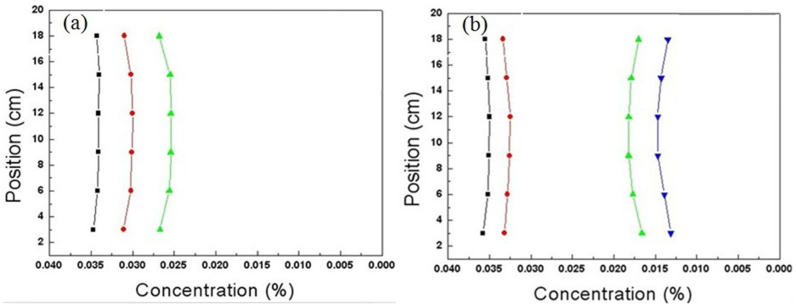
Measurement results of the time-dependent concentrations in the vertical straight lines of non-cloak. (a) The concentrations in three parallel lines. The parameters and the diffusion time are the same as that in Fig. 9a. (b) The concentrations in four parallel lines. The parameters and the diffusion time are the same as that in Fig. 10b.

For comparison, we measured the chloride ions concentration in three parallel straight lines of non-cloak (Fig. 9a). The concentrations of 18 test points are shown in Fig. 10a (the measurement time is 100 minutes), and the concentrations in a straight line are not equal. The concentrations of 24 test points (Fig. 9b) are shown in Fig. 10b (the measurement time is 200 minutes), and the concentrations in a straight line are not equal either. i.e., outside the outer circle, the iso-concentration lines are not parallel, i.e. the vertical parallel lines are not iso-concentration lines and the iso-concentration lines are not parallel. So this hollow cylinder is a non-cloak.

**Figure 11:**
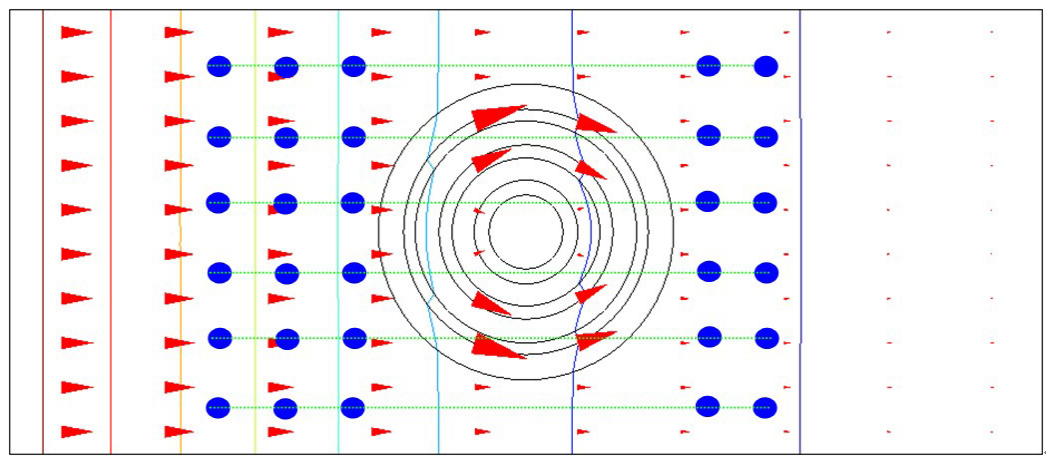
Test points of the time-dependant concentration gradients in horizontal lines. The diffusion time is 180 minutes; the horizontal lines represent diffusion flux lines. The measurement results are shown in Fig. 12.

**Figure 12:**
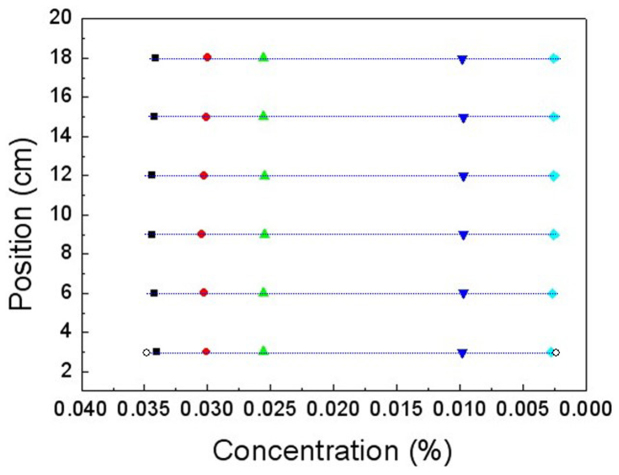
Measurement results of the time-dependant concentration gradients in horizontal lines. The parameters and diffusion time are the same as that in Fig. 11, the diffusion flux are parallel and equal.

By the same method, we measured the concentration gradients in the six horizontal lines (Fig. 11). The concentrations in the horizontal lines are shown in Fig. 12. The diffusion time is 180 minutes. Measurement results show that the concentration gradients are nearly equal and about 0.0027%/cm, so the horizontal lines are the diffusion flux lines.

**Figure 13:**
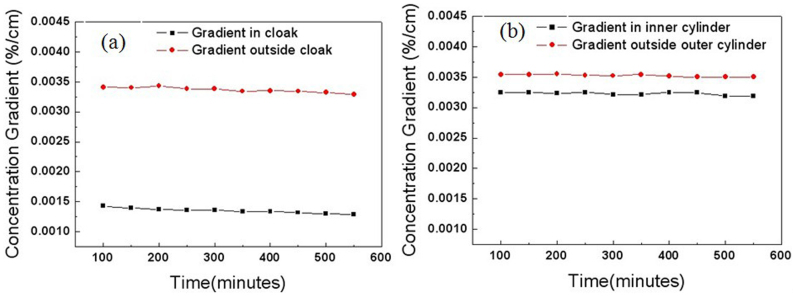
Measurement results of the time-dependent concentration gradients. (a) The parameters are the same as that in Fig. 4 (cloak). The red circles represent the concentration gradient between (−5.8 cm, 0) and (−4.2 cm, 0), and the black squares represent the concentration gradient between (−0.8 cm, 0) and (0.8 cm, 0). (b) The parameters are the same as that in Fig. 5 (non-cloak). The red circles represent the concentration gradients between (−5.6 cm, 0) and (−4.2 cm, 0), and the black squares represent the concentration gradients between (−0.8 cm, 0) and (0.8 cm, 0).

**Figure 14:**
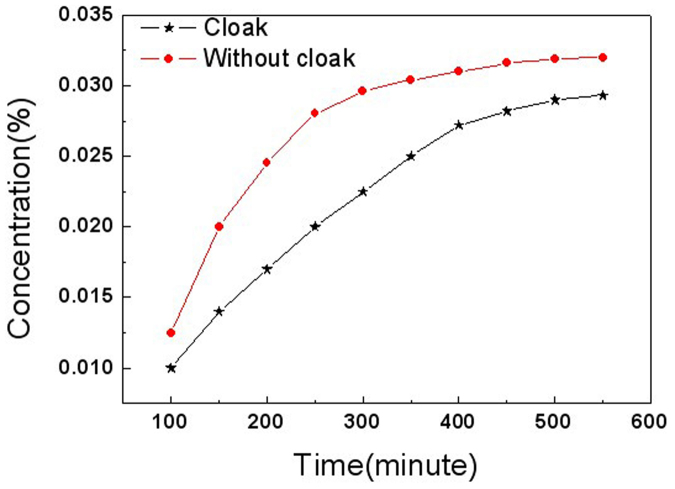
Measurement results of the time-dependent concentration in the center. The parameters of the red circles are the same as that in Fig. 13a (cloak), the parameters of the black five pointed star are the same as that in Fig. 13b (non-cloak). The concentrations in the center of the cloak are always smaller than those of the non-cloak even after 550 minutes.

Finally, we measured time-dependent concentration gradients in the inner circle and outside the outer circle. We fabricated ten identical diffusion cloak and ten identical hollow cylinders (non-cloak). In the inner circle, the test points are located at (−0.8 cm, 0) and (0.8 cm, 0), respectively, outside the outer circle, the test points are located at (−5.8 cm, 0) and (−4.2 cm, 0), respectively. The concentration gradients in the inner circle are always smaller than those outside the outer circle in the cloak even after 550 minutes (Fig. 13a). The concentration gradients in the inner circle are nearly equal to those outside the outer circle in the non-cloak (Fig. 13b). We also measured the time-dependent concentrations in the center of the cloak and non-cloak (Fig. 14). The concentrations in the center of the cloak are always smaller than those of the non-cloak even after 550 minutes. The six-layer cylinder we fabricated fulfills the conditions of cloak.

Guenneau^39^ theoretically studied two and three dimensional mass diffusion cloak in fluids, and numerically simulated multilayer nano-size reduced parameter cylinder and sphere mass diffusion cloak. In their work, they consider the convection–diffusion equation that is a parabolic partial differential equation combining the diffusion equation and the advection equation. The issue of convection is very importance for fluids diffusion. However, it is not necessary to consider the convection in the diffusion of chloride ions in solid concrete. Other diffusion of chemical element in containers such as cobalt, uranium can also be cloaked by our method. The prospects of mass diffusion cloak are bright in the construction field. If the materials come into being, it would prolong the life of construction materials. Meanwhile it also means a lot in chemical element diffusion research. Our works provide the theory and experiment guidance to protect concrete against chloride ions corrosion.

## Discussion

For an ideal mass diffusion cloak, the diffusion matters do not penetrate into the inner circle, thus a perfect diffusion protection is offered. The multilayer mass diffusion cloak is a simplified approximate cloak. The chloride ions can penetrate into the cloak region in long time diffusion. In a steady state, the cloak region eventually achieves a constant concentration. Therefore, the mass diffusion cloak can only provide a temporal diffusion protection rather than a permanent protection. Another case is that the diffusion matter chloride ions pollution would change the effective parameters of the cloaking materials; finally the chloride ions go into the inner cylinder after long time diffusion. In our experiment, we chose 0.5% NaCl solution. The chloride ions concentration in point P (−4.2 cm, 0) of Fig. 6 is less than 0.0400%, so we can ignore the influence of chloride ions pollution.

Chloride ions pollution will corrode rebars. Controlling chloride ions diffusion in concrete has a great significance in the field of building material. Concrete is composed of cement, aggregate and paste interfacial zone^40^,^41^, the larger the aggregate, the smaller the diffusion coefficient. Increasing or decreasing diffusion coefficient is a traditional method for protecting concrete^42^,^43^,^44^, Actually, this method is a “mass diffusion shielding”, just like electrostatic and magnetostatic shielding. We adapted the transformation optics method and designed six-layer mass diffusion cloak, the experiment demonstrated that the mass diffusion cloak can shield chloride ions and guide the chloride ions around the cloaked region. By mixing diffusion material, one can obtain various materials with a wide range diffusion coefficient, which is hardly available in the design of an electromagnet device.

## Methods

### Numerical simulation

We numerically simulate the steady and transient mass diffusion cloak by COMSOL Multiphysic software. The parameters fulfill Eq. (2) and Eq. (3).

### Fabricate samples

The samples are fabricated by six-layer concretes (ABACAD) and background media E, the concretes are made of cement, sand and glue mixture. The proportion of cement, sand and glue are A (1:3:0.02), B (1:3:0.30), C (1:3:0.20), D (1:3:0.10), E (1:3:0.08), respectively.

### Experiment measure

According to the simulation results of time-dependent mass diffusion cloak, we dug up a lot of small cylinders in the iso-concentration lines and concentration gradient lines, and measured the concentration of chloride ions by **NJCL** **Chloride Ions Concentration Fast Measurement Equipment**.

## Author Contributions

L.Z. wrote the paper and numerical stimulated. R.S. set up the experiment and revised the manuscript.
